# Supplementary material for: Comparison of gene set scoring methods for reproducible evaluation of multiple tuberculosis gene signatures
Source: bioRxiv. 2023 Jan 20:2023.01.19.520627. Preprint. [Version 1] doi: 10.1101/2023.01.19.520627 (PMC9882404; doi:10.1101/2023.01.19.520627)

Supplementary Table 1. Summary of AUC and 95% CI results from gene signature's discovery/training study. The original publication section presented the AUC and 95% CI for each signature from their publication. The Warsinske et al. subsection showed the results that have been re-evaluated using the original models functionality that had been implemented in the TBSignatureProfiler, and the ssGSEA section presented the performance of each gene signature using ssGSEA from TBSignatureProfiler (Courtesy to Warsinske et al. work; ATB: active tuberculosis).

Signature	Clinical Comparison	Dataset Compared	Original Publication	Warsinske et al.	TBSignatureProfiler	
			AUC (95% CI)	AUC (95% CI)	AUC (95% CI) Warsinske et al.	AUC (95%CI) ssGSEA
Sweeney_OD_3	ATB vs. HCs	GSE19491✧	0.96 (0.94 - 0.98)	0.96 (0.94 - 0.98)	0.95 (0.91 - 0.99)	0.96 (0.91 - 0.99)
		GSE42834✧	1.00 (1.00-1.00)	1.00 (1.00-1.00)	1.00 (1.00 - 1.00)	0.92 (0.83 - 0.98)
	ATB vs. LTBI	GSE19491✧	0.93 (0.91 - 0.95)	0.93 (0.91-0.95)	0.92 (0.87 - 0.97)	0.93 (0.87 - 0.97)
		GSE37250	0.93 (0.91 - 0.94)	0.93 (0.91-0.94)	0.93 (0.90 - 0.95)	0.90 (0.86 - 0.93)
	ATB vs. OD	GSE19491✧	0.92 (0.89 - 0.94)	0.92 (0.89-0.94)	0.90 (0.84 - 0.95)	0.92 (0.88 - 0.97)
		GSE37250	0.87 (0.85 - 0.89)	0.87 (0.85-0.89)	0.87 (0.83 - 0.91)	0.83 (0.78 - 0.87)
		GSE42834✧	0.84 (0.80 - 0.88)	0.84 (0.80-0.88)	0.84 (0.79 - 0.90)	0.78 (0.67 - 0.85)
Jacobsen_3	ATB vs. LTBI	GSE19491✧	N/A	0.93 (0.91-0.95)	0.93 (0.88 - 0.97)	0.79 (0.71 - 0.86)
LauxdaCosta_OD_3	ATB vs. OD	GSE42834✧	0.95* (0.88-1.00*)	1.00 (1.00-1.00)	1.00 (1.00-1.00)	0.82 (0.76 - 0.88)
Maertzdorf_4	ATB vs. HCs	GSE74092	0.98 (0.96 - 1.00)	0.99 (0.99-1.00)	1.00 (1.00-1.00)	0.71 (0.64 - 0.79)
Sambarey_HIV_10	ATB vs. (LTBI & HCs & OD)	GSE37250*	N/A	0.89 (0.87-0.91)	0.89 (0.85 - 0.91)	0.79 (0.75 - 0.82)
Verhagen_10	ATB vs. (LTBI & HCs)	GSE41055	N/A	1.00 (1.00-1.00)	1.00 (1.00-1.00)	0.85 (0.66 - 0.97)
Maertzdorf_15	ATB vs. HC	GSE74092	0.99 (0.97 - 1.00)	1.00 (1.00-1.00)	1.00 (1.00 - 1.00)	0.95 (0.92 - 0.97)
Leong_24	ATB vs. LTBI	GSE101705	0.98 (0.98 - 0.98)	1.00 (1.00-1.00)	1.00 (1.00 - 1.00)	0.95 (0.87 - 1.00)
Kaforou_27	ATB vs. LTBI	GSE19491✧	0.98 (0.95 - 1.00)	0.95 (0.93-0.97)	0.96 (0.92 - 0.99)	0.95 (0.90 - 0.98)
Anderson_42	ATB vs. LTBI	GSE39940	0.98 (0.95 - 1.00)	0.97 (0.96-0.98)	0.98 (0.95 - 0.99)	0.93 (0.88 - 0.96)
Kaforou_OD_44	ATB vs. OD	GSE19491✧	0.95 (0.89 - 0.99)	0.91 (0.89-0.94)	0.93 (0.90 - 0.96)	0.69 (0.60 - 0.77)
Anderson_OD_51	ATB vs. OD	GSE39940	0.86 (0.77 - 0.94)	0.89 (0.87-0.91)	0.90 (0.87 - 0.94)	0.80 (0.75 - 0.85)
Kaforou_OD_53	ATB vs. (LTBI & HCs & OD)	GSE19491✧	N/A	0.92 (0.89-0.94)	0.92 (0.88 - 0.96)	0.71 (0.63 - 0.77)

Supplementary Table 1 (Continued)

Signature	Clinical Comparison	Dataset Compared	Original Publication	Warsinske et al.	TBSignatureProfiler	
			AUC (95% CI)	AUC (95% CI)	AUC (95% CI) Warsinske et al.	AUC (95%CI) ssGSEA
Berry_OD_86	ATB vs. LTBI	GSE19491✧	N/A	0.97 (0.96-0.99)	0.96 (0.92 - 0.98)	0.90 (0.85 - 0.95)
	ATB vs. HCs	GSE19491✧	N/A	1.00 (1.00-1.00)	0.99 (0.98 - 1.00)	0.89 (0.84 - 0.94)
Bloom_OD_144	ATB vs. (OD & HCs)	GSE42834✧	0.91 (Not available)	0.99 (0.98-1.00)	0.97 (0.94 - 1.00)	0.80 (0.71 - 0.87)
Berry_393	ATB vs. LTBI	GSE19491✧	N/A	0.97 (0.96-0.99)	0.96 (0.93 - 0.99)	0.94 (0.89 - 0.97)
	ATB vs. HCs	GSE19491✧	N/A	0.99 (0.98-1.00)	0.99 (0.97 - 1.00)	0.95 (0.91 - 0.98)
Leong_RISK_29	Progressor vs. Non-progressor	GSE79362 (Baseline)	N/A	N/A	0.96 (0.93 - 0.99)	0.56 (0.51 - 0.69)
Zak_RISK_16	Progressor vs. Non-progressor	GSE79362 (Total Time Period)	0.74 (0.73 - 0.76)	N/A	0.72 (0.61 - 0.82)	0.77 (0.62 - 0.87)
Suliman_RISK_4 (Site-Specific)	Progressor vs. Non-progressor	GSE94438	0.67 (0.57-0.77)	N/A	0.71 (0.65 - 0.77)	0.61 (0.55 - 0.67)

* Original training studies were not available for the signatures; ✧ Superseries of multiple datasets

bioRxiv preprint doi: https://doi.org/10.1101/2023.01.19.520627; this version posted January 20, 2023. The copyright holder for this preprint (which was not certified by peer review) is the author/funder, who has granted bioRxiv a license to display the preprint in perpetuity. It is made available under aCC-BY 4.0 International license.

Supplementary Figure 1. The distribution of AUC values for 19 TB gene signatures across 24 studies using ssGSEA **(A)**, GSVA **(B)**, PLAGE **(C)**, Zscore **(D)**, and Singscore unidirectional version **(E)**. Datasets had the same order as those from Figure 1.

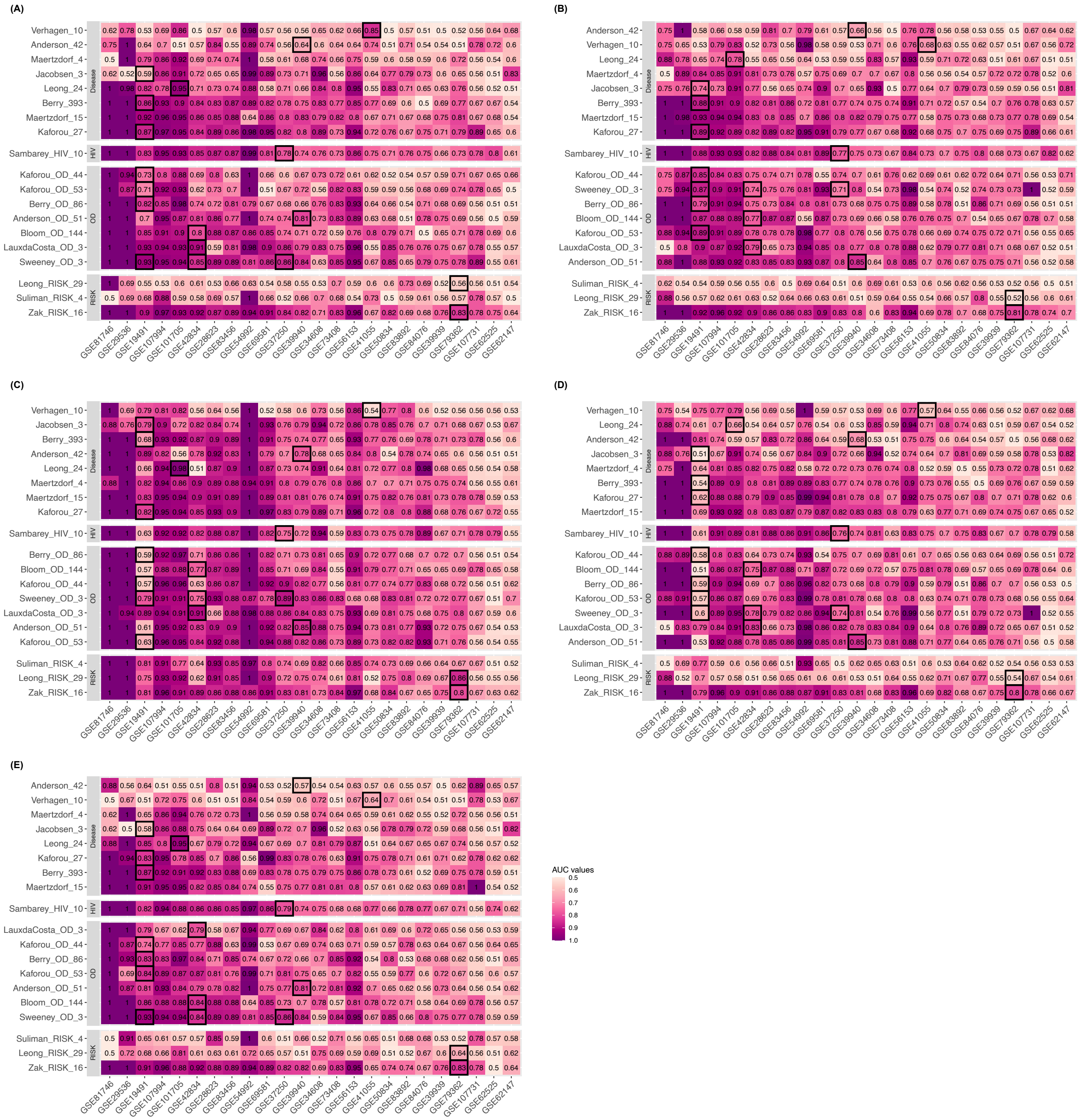

Supplementary Figure 2. The distribution of AUC values for **upregulated and downregulated subsets** of 13 TB gene signatures across 24 studies using ssGSEA **(A)**, GSVA**(B)**, and Singscore bidirectional version **(C)**. Datasets had the same order as those from Figure 1.

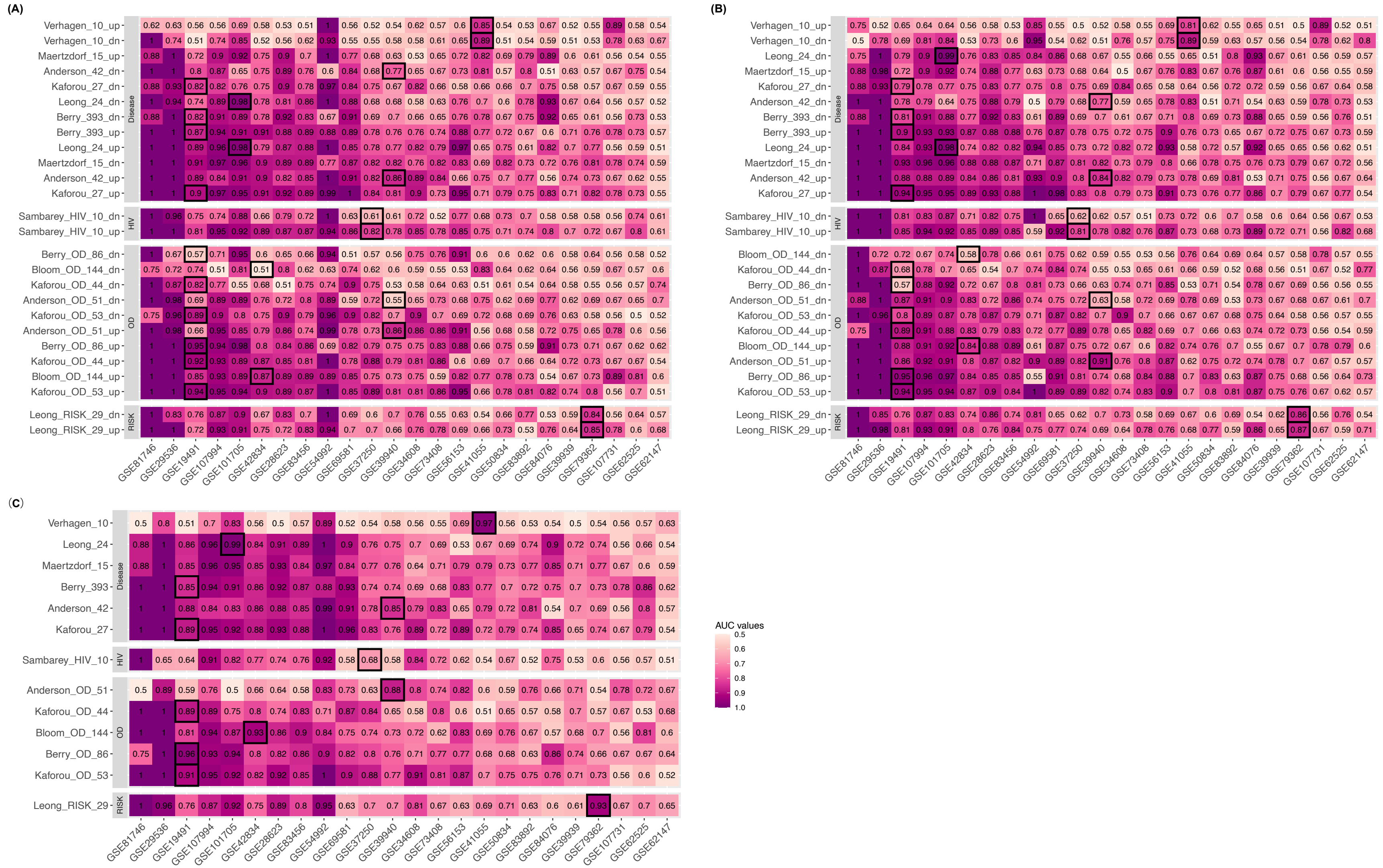

bioRxiv preprint doi: https://doi.org/10.1101/2023.01.19.520627; this version posted January 20, 2023. The copyright holder for this preprint (which was not certified by peer review) is the author/funder, who has granted bioRxiv a license to display the preprint in perpetuity. It is made available under aCC-BY 4.0 International license.

Supplementary Figure 3. The distribution of AUC values for TB gene signatures across 24 studies using the original models and gene set scoring methods in the form of ridge plot. Gene signatures were ordered based on the median AUCs given by the original models. **(A)** Comparison of AUC distributions given by ssGSEA (evaluated on the upregulated subsets of the gene signatures), PLAGE, and the original models. **(B)** Comparison of AUC distributions given by five gene set scoring methods (ssGSEA, PLAGE, GSVA, Singscore, Zscore) and the original models. **(C)** Comparison of AUC distributions given by ssGSEA (evaluated on the upregulated and downregulated subsets of gene signatures), GSVA (evaluated on the upregulated and downregulated subsets of gene signatures), and the original models.

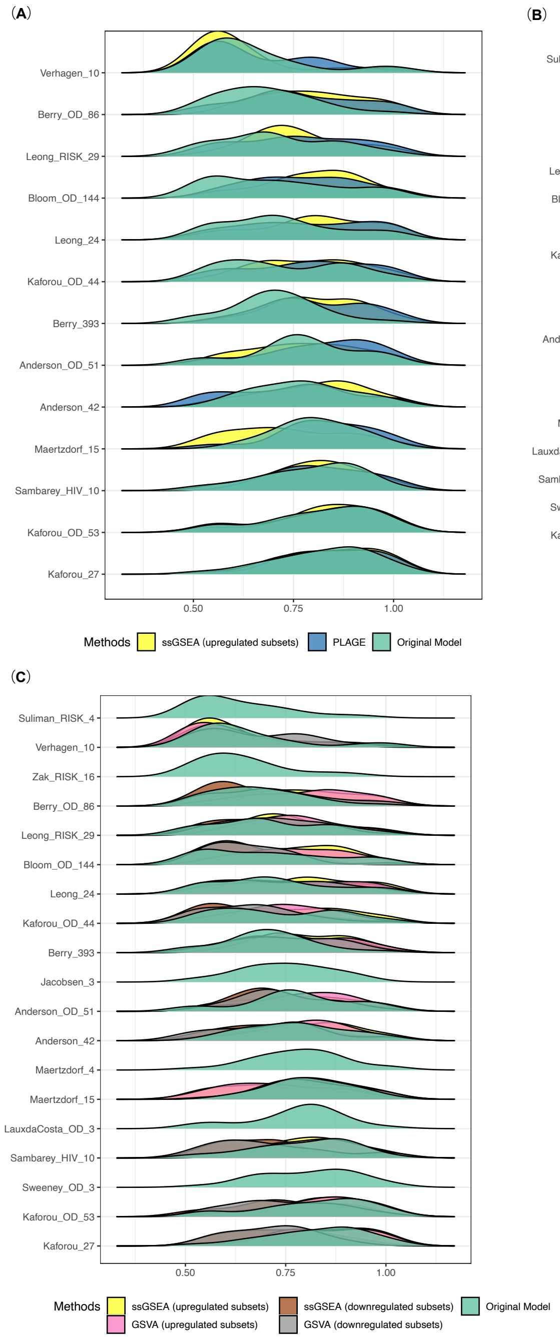

Supplementary Figure 4. Spearman's rank correlation versus AUC difference for studies with sample size larger than 40 and computed AUC values greater than 0.8 based on the results given by original models and ssGSEA **(A)**, original models and PLAGE **(B)**, original models and GSVA **(C)**, original models and Zscore **(D)**, original models and Singscore (unidirectional version) **(E)**. Overlapping points indicate the same dataset(s).

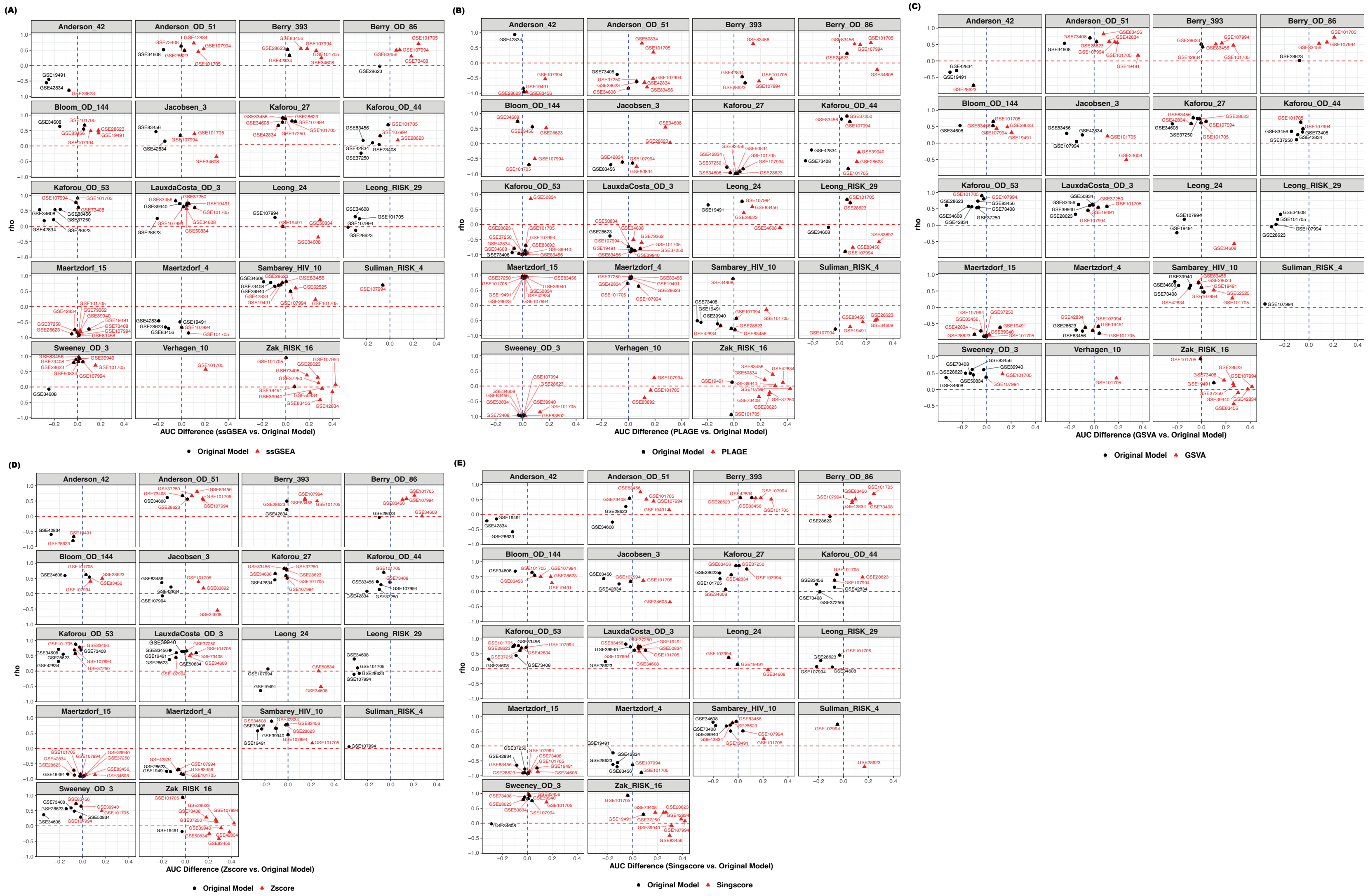

bioRxiv preprint doi: https://doi.org/10.1101/2023.01.19.520627; this version posted January 20, 2023. The copyright holder for this preprint (which was not certified by peer review) is the author/funder, who has granted bioRxiv a license to display the preprint in perpetuity. It is made available under aCC-BY 4.0 International license.

Supplementary Figure 5. Spearman's rank correlation versus AUC difference for studies with sample size larger than 40 and computed AUC greater than 0.8 based on results given by original models and ssGSEA (evaluated with upregulated subsets) **(A)**, original models and ssGSEA (evaluated with downregulated subsets) **(B)**, original models and GSVA (evaluated with upregulated subsets) **(C)**, original models and GSVA (evaluated with downregulated subsets) **(D)**, original models and Singscore (bidirectional version) **(E)**. Overlapping points indicate the same dataset(s).

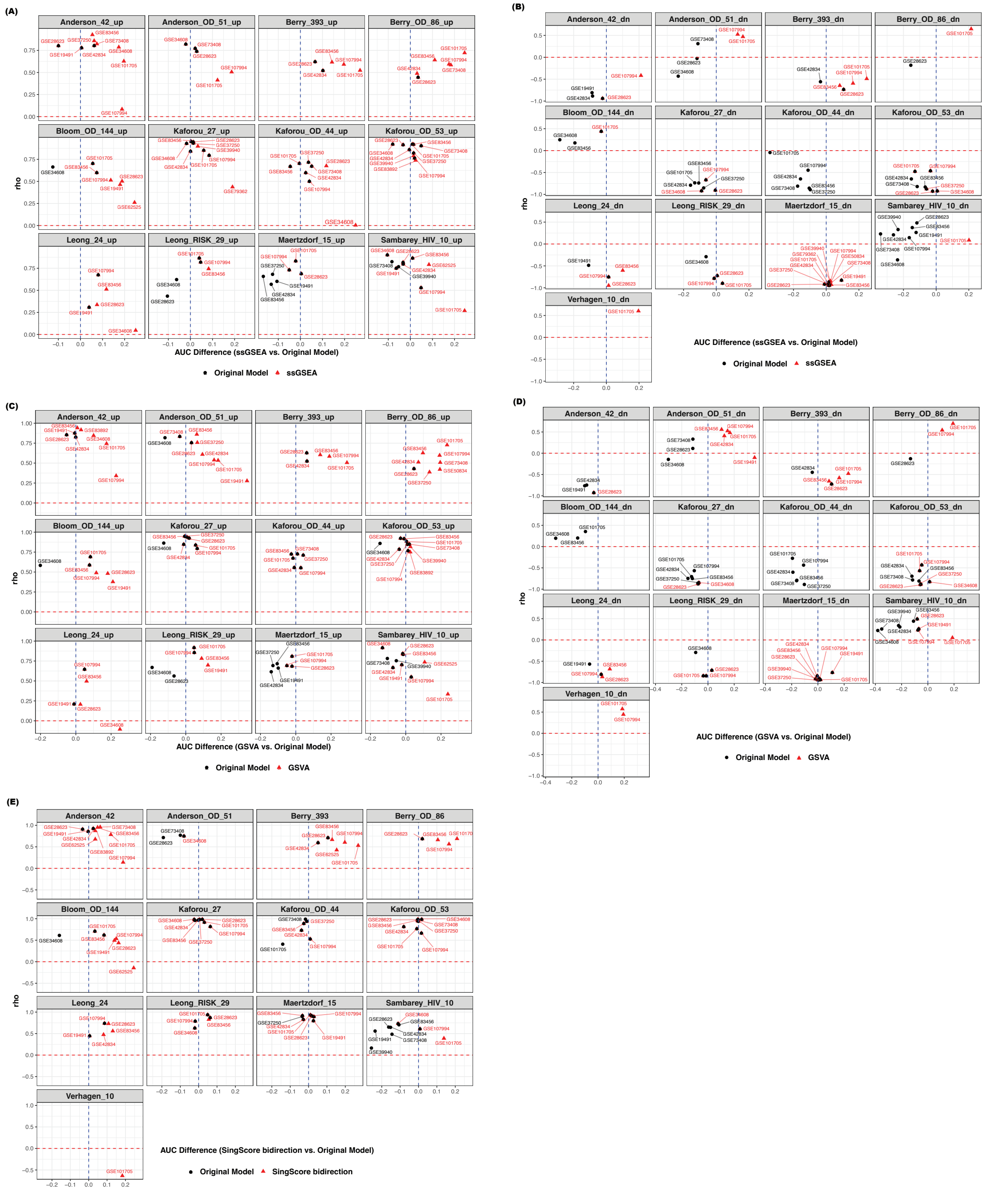

Supplement: Supplementary file 1 [file NIHPP2023.01.19.520627v1-supplement-2.pdf]
